# Variation in quantitative CT air trapping in heavy smokers on repeat CT examinations

**DOI:** 10.1007/s00330-012-2526-y

**Published:** 2012-06-14

**Authors:** Onno M. Mets, Ivana Isgum, Christian P. Mol, Hester A. Gietema, Pieter Zanen, Mathias Prokop, Pim A. de Jong

**Affiliations:** 1 Radiology, University Medical Center Utrecht, Heidelberglaan 100, Postbus 85500, 3508 GA Utrecht, The Netherlands; 2 Image Sciences Institute, University Medical Center Utrecht, Heidelberglaan 100, 3508 GA Utrecht, The Netherlands; 3 Pulmonology, University Medical Center Utrecht, Heidelberglaan 100, 3508 GA Utrecht, The Netherlands; 4 Radiology, Radboud University Nijmegen Medical Centre, Geert Grooteplein-Zuid 10, 6525 GA Nijmegen, The Netherlands

**Keywords:** Quantitative computed tomography, Airway remodeling, Small airways disease, Tobacco smoking, Chronic obstructive pulmonary disease

## Abstract

**Objectives:**

To determine the variation in quantitative computed tomography (CT) measures of air trapping in low-dose chest CTs of heavy smokers.

**Methods:**

We analysed 45 subjects from a lung cancer screening trial, examined by CT twice within 3 months. Inspiratory and expiratory low-dose CT was obtained using breath hold instructions. CT air trapping was defined as the percentage of voxels in expiratory CT with an attenuation below −856 HU (EXP_−856_) and the expiratory to inspiratory ratio of mean lung density (E/I-ratio_MLD_). Variation was determined using limits of agreement, defined as 1.96 times the standard deviation of the mean difference. The effect of both lung volume correction and breath hold reproducibility was determined.

**Results:**

The limits of agreement for uncorrected CT air trapping measurements were −15.0 to 11.7 % (EXP_−856_) and −9.8 to 8.0 % (E/I-ratio_MLD_). Good breath hold reproducibility significantly narrowed the limits for EXP_−856_ (−10.7 to 7.5 %, *P* = 0.002), but not for E/I-ratio_MLD_ (−9.2 to 7.9 %, *P* = 0.75). Statistical lung volume correction did not improve the limits for EXP_−856_ (−12.5 to 8.8 %, *P* = 0.12) and E/I-ratio_MLD_ (−7.5 to 5.8 %, *P* = 0.17).

**Conclusions:**

Quantitative air trapping measures on low-dose CT of heavy smokers show considerable variation on repeat CT examinations, regardless of lung volume correction or reproducible breath holds.

**Key Points:**

• *Computed tomography quantitatively measures small airways disease in heavy smokers*.

• *Measurements of air trapping vary considerably on repeat CT examinations*.

• *Variation remains substantial even with reproducible breath holds and lung volume correction*.

## Introduction

Chronic obstructive pulmonary disease (COPD) is a major cause of morbidity and mortality worldwide, and is projected to become one of the leading causes of death in the world in the coming decades [1, 2]. It is now thought that the disease starts long before obstruction is measurable on spirometry by narrowing and disappearance of small airways, before the onset of emphysematous destruction which eventually results in deterioration of lung function [3]. This sequence of events makes the evaluation of small airways disease highly interesting for measuring disease progression in the early stages of smoking-induced lung disease. The hallmark of small airways dysfunction on imaging is air trapping, and this can be evaluated in expiratory computed tomography (CT). Air trapping can be automatically quantified by CT, which makes it suitable to study disease progression in large cohort studies [4, 5], lung cancer screening examinations [6, 7] and drug trials. However, to be able to detect disease progression or effects of therapy, variation between examinations should be within acceptable limits as disease progression is defined as an increase above the upper limit of such variation. The variation with the limits of agreement for repeat CT studies in emphysema is known [8, 9]; however, to our knowledge no information is available on the variation of CT air trapping quantification. Therefore, the objective of this study was to determine the variation in two commonly used quantitative CT air trapping measures in current and former heavy smokers.

## Methods

### Subjects

Subjects were participants in the Dutch-Belgian Lung Cancer Screening (NELSON) trial, a population-based randomised lung cancer screening trial [7]. NELSON subjects are current or former (<10 year) heavy smokers aged between 50 and 75 years, with a smoking history of at least 16 cigarettes/day for 25 years or at least 11 cigarettes/day for 30 years (i.e. >16.5 pack-years). Patient characteristics and smoking history were collected at baseline. Exclusion criteria for participating in the trial were a self-reported moderate or bad health with inability to climb two flights of stairs, a recent chest CT, current or past cancer and a body weight greater than or equal to 140 kg [7]. The trial was approved by the Dutch Ministry of Health and by the local ethical review board. Written informed consent was obtained from each participant. In one centre, expiratory CT was added to the screening protocol during the third screening round for a prospective side-study into COPD. Since the lung cancer screening trial participants are current and former heavy smokers with no or mainly mild COPD [10], it enabled us to study the variation between repeat CT examinations for quantitative CT air trapping in a population with early stages of disease. The adjustment of the screening protocol was separately approved by the local ethical review board of our hospital.

For the present study, we retrospectively included all consecutive participants who received short-term repeat CT imaging in inspiration and expiration between July 2007 and September 2008 for an indeterminate nodule (approximately 6 weeks interval between CTs, *n* = 70). No formal power calculation was performed. We excluded four subjects due to failure of the expiratory CT, six due to lung segmentation errors (see next paragraph) and 15 due to differences in CT protocol at follow-up (different kVp in 13 and different CT equipment in 2 subjects). The final study population therefore consisted of 45 subjects, with two paired inspiratory and expiratory CT examinations obtained on the same CT system, with the same protocol. Pulmonary function data are not available for this study population, given that only a random subset of all screening trial participants received pulmonary function testing due to the lung cancer screening study design.

### Computed tomography and quantitative analysis

All subjects underwent volumetric CT of the chest in inspiration and end-expiration, after standardised breathing instructions. All CTs were acquired with 16 × 0.75 mm collimation (Brilliance 16P; Philips Medical Systems, Cleveland OH, USA). Settings were adjusted to body weight: 120 kVp (≤80 kg) or 140 kVp (>80 kg) both at 30 mAs for inspiratory CT, and 90 kVp (≤80 kg) or 120 kVp (>80 kg) both at 20 mAs for expiratory CT. A combined inspiratory and expiratory CT yielded an estimated effective dose of 1.2–2.0 mSv, of which 0.3–0.65 mSv is accounted for by the expiration CT. Images with section thickness of 1.0 mm at 0.7 mm increment were reconstructed from lung bases to lung apices using a smooth reconstruction kernel (B-filter; Philips Medical Systems, Cleveland OH, USA), according to the protocol.

The lungs were automatically segmented using dedicated software [11], and a noise reduction filter was applied to decrease the influence of noise on the quantitative measurements [12]. Briefly, the lungs were segmented from the chest wall, mediastinum, diaphragm and airways in both inspiratory and expiratory CT images. Total volume and attenuation of all voxels included in the lung segmentation was calculated and a density histogram created, from which the quantitative CT air trapping measures were extracted. Lung segmentation was visually checked in all CT pairs of each participant. The extent of CT air trapping was defined as the percentage of voxels in expiratory CT with an attenuation below −856 HU (EXP_−856_) [5] and as the expiratory to inspiratory ratio of mean lung density (E/I-ratio_MLD_) [13], which are both currently available techniques to quantify air trapping in COPD on CT. Quantitative results are presented as percentage.

The influence of lung volume on densitometry has previously been reported [14–16]. Given that differences in inspiratory and expiratory volume will lead to differences in lung density we evaluated the effect of lung volume correction on the CT air trapping measures, as was proposed to be essential for quantitative lung densitometric analyses [17, 18]. Additionally, we arbitrarily subdivided the subjects into two groups to determine the susceptibility of the CT air trapping measures for differences in inspiratory and expiratory volume; one subgroup with a difference in expiratory volume (EXP_−856_) or exhaled lung volume (E/I-ratio_MLD_) on repeat examination in the outer quartiles (i.e. Q1 and Q4), and one subgroup within the interquartile range around the median (i.e. Q2 and Q3). These subgroups are further referred to as inferior and superior breath hold reproducibility, respectively.

### Quality control

**Fig. 1:**
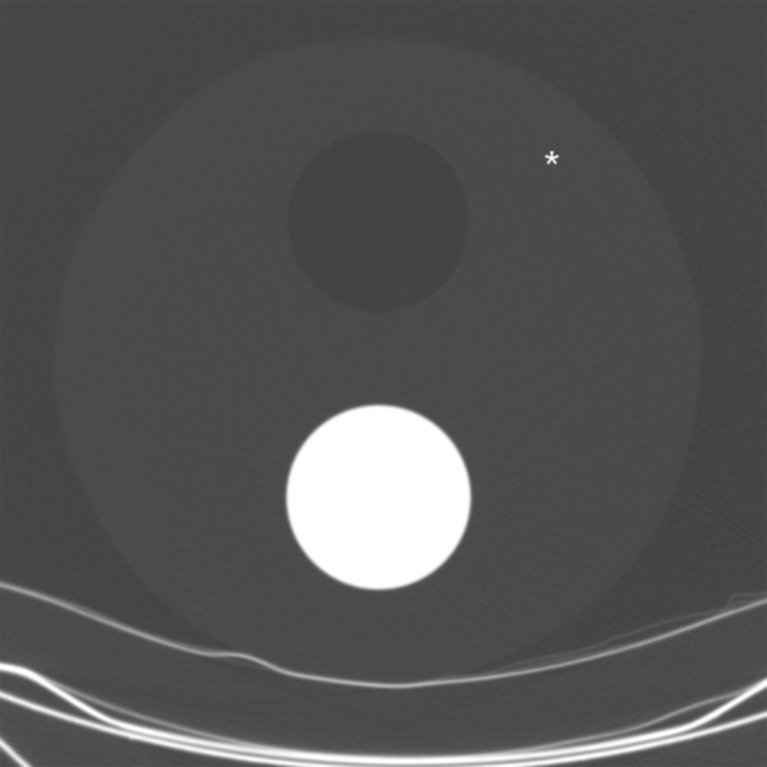
Quality control phantom used to monitor the CT numbers over time. The phantom consists of a 32-cm-wide foam body containing two cylinders filled with air and plastic, respectively. Mean CT numbers were determined using a manually placed region of interest, drawn at a fixed location in the foam body (see *asterisk*)

In addition to standard calibration procedures performed according to the manufacturer’s guidelines, a quality control phantom was imaged at each data acquisition session to monitor CT numbers during the period of data collection. The 32-cm-wide phantom consists of a foam body, which mimics emphysematous lung parenchyma, and further includes two separate cylinders filled with air and plastic, respectively (Fig. 1). The phantom was imaged with the same protocol as was applied to the participants (120 kVp, 30 mAs, B-filter). One observer with 2 years of experience in thoracic CT manually placed circular regions of interest with a diameter of 20 mm at a fixed location in the foam body of the phantom; from these regions the average CT number was calculated.

### Statistical analysis

Reproducibility of CT air trapping measures between the two visits was assessed by the concordance correlation coefficient (*p*_c_), which takes into account both the correlation and the distance to the line of identity [19]. Differences between the two visits were calculated by subtracting the values from the repeat CT from the values from the baseline CT (i.e. Δ = CT1 − CT2). The resulting difference was plotted against the mean of both results, using the Bland–Altman approach [20]. The limits of agreement were defined as ±1.96 times the standard deviation of the mean difference. The limits of agreement (i.e. heteroscedasticity) of uncorrected and volume corrected CT air trapping values within subjects were tested for significance according to Sandvik and Olsson’s method [21]. The heteroscedasticity of CT air trapping values between subjects with superior and inferior breath hold reproducibility was tested with Levene’s test [22].

All statistical analyses were performed using SPSS software v15.0 (SPSS Inc, Chicago, Illinois, USA). Bland–Altman plots and concordance correlation coefficients were calculated using MedCalc v11.3.8.0, Mariakerke Belgium. A *P* value less than 0.05 was considered statistical significant. Data given are median (25th–75th percentile), unless indicated otherwise.

## Results

### Study population

The study population consisted of 45 subjects (44 male), with a mean ± standard deviation (SD) age of 64.1 ± 5.1 years. Repeat CT was performed after 6.7 (6.1–7.0) weeks. Study population characteristics are summarised in Table 1.

**Table 1:** Study population characteristics

Male gender, *n* (%)	44 (98 %)
Age in years, mean ± SD	64.1 ± 5.1
Height in cm, mean ± SD^a^	180 ± 7
Smoking status^a^	
Current smoker, *n* (%)	27 (60)
Ex smoker, *n* (%)	14 (31)
Pack-years^a^, median (interquartile range)	37 (30–49)
Follow-up period in weeks, median (interquartile range)	6.7 (6.1–7.0)

^a^Missing data in 4 cases

### Quality control

Mean CT numbers of the foam were −966 ± 2.1 HU during the study. The standard deviation of 2.1 HU is well within the acceptable range reported by the vendor (0–4 HU).

### Variability in quantitative CT assessment of air trapping

**Fig. 2:**
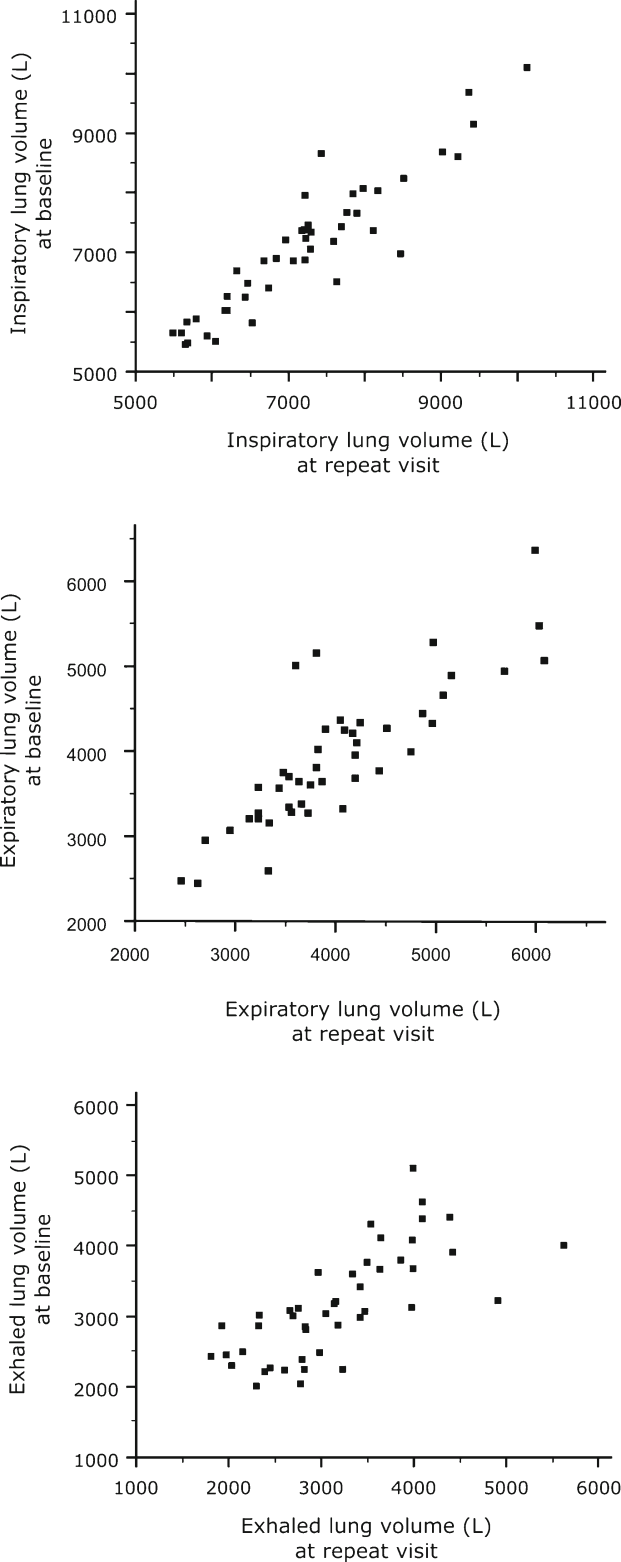
Relationship between inspiratory (*top*), expiratory (*middle*) and exhaled (*bottom*) lung volume in baseline and repeat CT

Inspiratory and expiratory volumes generally showed good repeatability; the median (interquartile range) absolute differences in total lung volume were −145 mL (−303 to 149) for inspiratory volume, −116 mL (−424 to 157) for expiratory volume, and 26 mL (−385 to 393) for exhaled volume. The association between the two acquisitions is illustrated in Fig. 2.

At baseline, CT air trapping measures ranged from 0.5 to 59.1 % for EXP_−856_, and from 64.5 to 95.1 % for E/I-ratio_MLD_. At repeat CT examination, this was 0.9 to 67.5 % for EXP_−856_, and 70.7 to 96.5 % for E/I-ratio_MLD_. CT air trapping between the two acquisitions showed a concordance correlation coefficient of 0.886 (EXP_−856_) and 0.741 (E/I-ratio_MLD_). The difference in CT air trapping between the two acquisitions ranged from −13.3 to 19.6 % for EXP_−856_, and from −13.9 to 9.9 % for E/I-ratio_MLD_.

The difference in CT air trapping in subjects with superior breath hold reproducibility ranged from −12.8 to 7.6 % for EXP_−856_, and from −13.9 to 5.4 % for E/I-ratio_MLD_. The quantitative results in subjects with inferior and superior breath hold reproducibility are further summarised in Table 2. After lung volume correction was applied, the difference in CT air trapping between the two acquisitions ranged from −12.6 to 14.9 % for EXP_−856_, and from −9.5 to 9.5 % for E/I-ratio_MLD_. The quantitative results for uncorrected and volume-corrected CT air trapping measures are summarised in Table 3.

**Table 2:** Quantitative CT measures of air trapping according to breath hold reproducibility

	EXP_−856_ (%) inferior reproducibility^a^	EXP_−856_ (%) superior reproducibility^b^	E/I-ratio_MLD_ (%) inferior reproducibility^a^	E/I-ratio_MLD_ (%) superior reproducibility^b^
	(*n* = 22)	(*n* = 23)	(*n* = 22)	(*n* = 23)
Baseline CT				
Median	14.0	7.7	87.5	85.2
IQR	5.3–23.3	1.6–16.4	80.3–90.9	79.3–87.2
Range	0.5–59.1	0.6–39.1	64.5–95.1	67.6–90.2
Repeat CT				
Median	15.4	9.4	87.1	85.2
IQR	4.6–27.0	2.3–20.9	83.1–92.0	81.6–88.2
Range	2.0–67.5	0.9–43.9	77.4–96.5	70.7–91.2
Difference				
Mean	−1.7	−1.6	−1.1	−0.6
Limits of agreement^c^	−18.7 to 15.3	−10.7 to 7.5	−10.5 to 8.3	−9.2 to 7.9
	*P* = 0.002		*P* = 0.75	

*EXP*_*−856*_ CT air trapping score as percentage of lung voxels below −856 HU in expiratory CT, *E/I-ratio*_*MLD*_ CT air trapping score as expiratory to inspiratory ratio of mean lung density, *IQR* interquartile range

^a^Defined as a difference of expiratory volume (EXP_−856_) or exhaled lung volume (E/I-ratio_MLD_) outside the interquartile range on repeat examination

^b^Defined as a difference of expiratory volume (EXP_−856_) or exhaled lung volume (E/I-ratio_MLD_) within the interquartile range on repeat examination

^c^Defined as ±1.96 times the standard deviation of the mean difference

**Table 3:** Quantitative CT measures of air trapping in 45 subjects at baseline and repeat CT

	EXP_−856_ (%)	EXP_−856_ (%) volume corrected	E/I-ratio_MLD_ (%)	E/I-ratio_MLD_ (%) volume corrected
Baseline CT				
Median	10.8	12.8	85.6	85.2
IQR	3.5–22.8	4.8–23.5	80.2–89.2	80.5–88.9
Range	0.5–59.1	−4.8 to 53.6	64.5–95.1	68.7–93.8
Repeat CT				
Median	11.7	14.3	85.6	85.9
IQR	2.7–25.0	4.8–24.3	81.7–88.7	81.1–89.1
Range	0.9–67.5	1.1–63.9	70.7–96.5	71.0–97.1
Difference				
Mean	−1.6	−1.8	−0.9	−0.9
Limits of agreement^a^	−15.0 to 11.7	−12.5 to 8.8	−9.8 to 8.0	−7.5 to 8.8
	*P* = 0.12		*P* = 0.17	

*EXP*_*−856*_ CT air trapping score as percentage of lung voxels below −856 HU in expiratory CT, *E/I-ratio*_*MLD*_ CT air trapping score as expiratory to inspiratory ratio of mean lung density, *IQR* interquartile range

^a^Defined as ±1.96 times the standard deviation of the mean difference

**Fig. 3:**
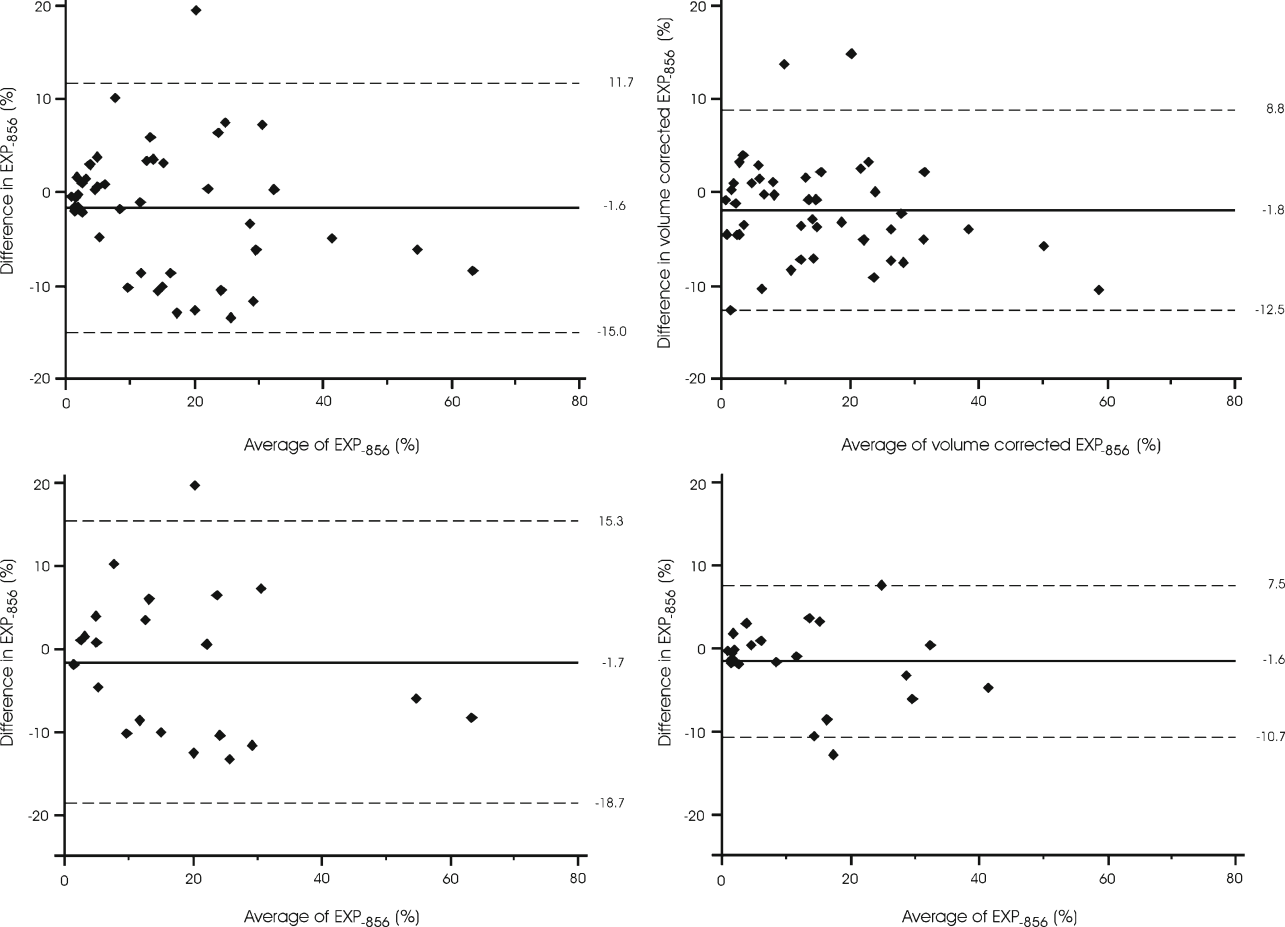
Variation in CT air trapping (EXP_−856_) on repeat CT examinations. The *left upper graph* shows the uncorrected values for the complete group. The *right upper graph* shows the volume corrected values for the complete group. The *lower graphs* show the uncorrected values for subjects with inferior (*left*) and superior (*right*) breath hold reproducibility, defined as difference of expiratory lung volume outside or within the interquartile range around the mean, respectively. The *x-axes* show the means of CT air trapping measurements at both acquisitions, and the *y-axes* show the differences between the measurements. The *solid lines* represent the mean difference, whereas the *dashed lines* represent the limits of agreement

**Fig. 4:**
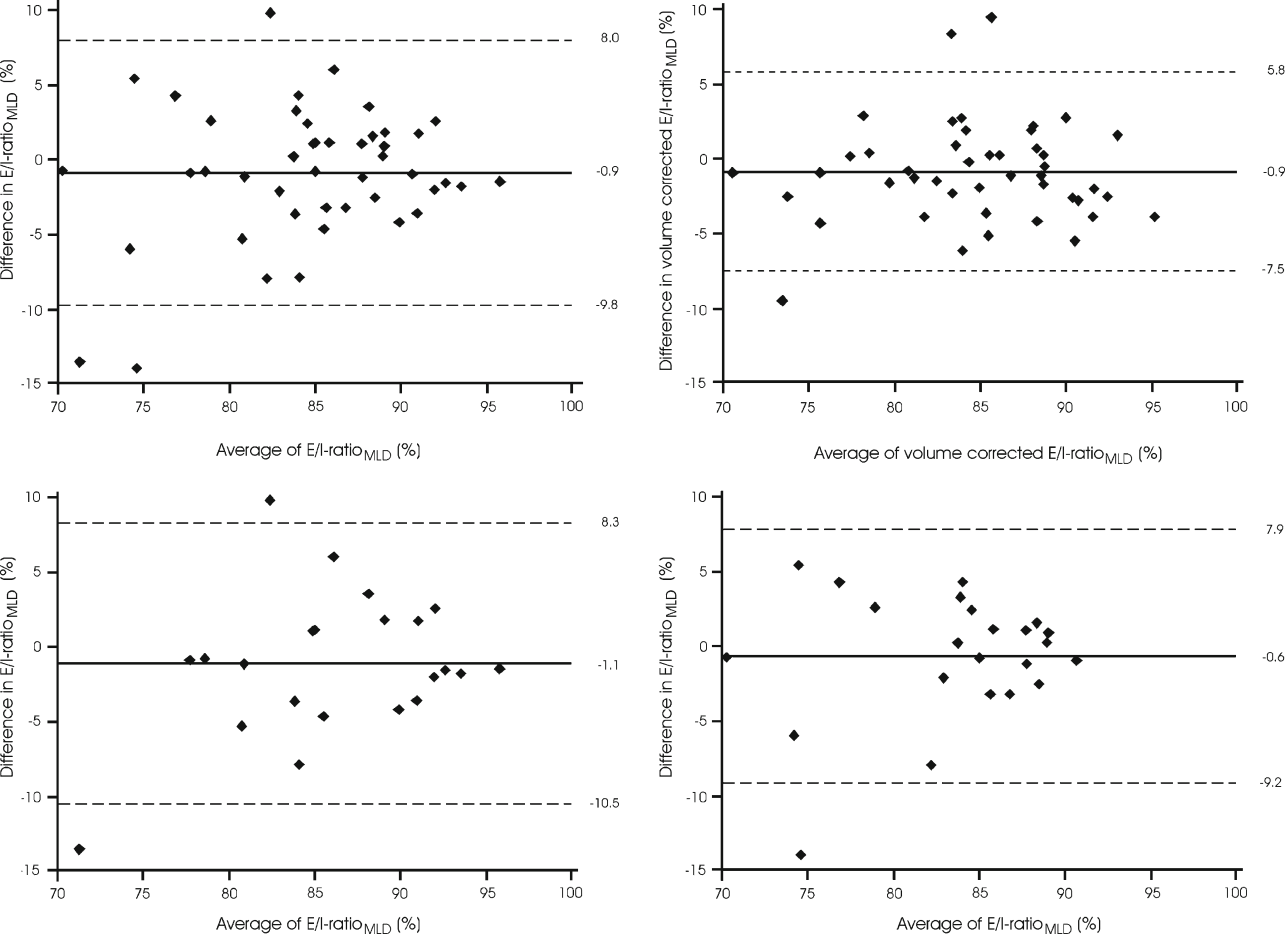
Variation in CT air trapping (E/I-ratio_MLD_) on repeat CT examinations The *left upper graph* shows the uncorrected values for the complete group. The *right upper graph* shows the volume corrected values for the complete group. The *lower graphs* show the uncorrected values for subjects with inferior (*left*) and superior (*right*) breath hold reproducibility, defined as difference between CT examinations of exhaled lung volume outside or within the interquartile range around the median, respectively. The *x-axes* show the means of CT air trapping measurements at both acquisitions, and the *y-axes* show the differences between the measurements. The *solid lines* represent the mean difference, whereas the *dashed lines* represent the limits of agreement

The variation in CT air trapping measures on repeat CT examinations is summarised in Figs. 3 and 4. As shown, the limits of agreement for the uncorrected quantitative CT air trapping measures were −15.0 to 11.7 % for EXP_−856_ and −9.8 % to 8.0 % for E/I-ratio_MLD_. After application of lung volume correction, this changed to −12.5 to 8.8 % (EXP_−856_, *P* = 0.12) and −7.5 to 5.8 % (E/I-ratio_MLD_, *P* = 0.17). In subjects with superior breath hold reproducibility, the limits of agreement were −10.7 to 7.5 % (EXP_−856_, *P* = 0.002) and −9.2 to 7.9 % (E/I-ratio_MLD_, *P* = 0.75).

## Discussion

We report the limits of agreement in quantitative assessment of CT air trapping in current and former heavy smokers. Knowledge on these limits is mandatory to be able to judge whether differences between two acquisitions are caused by actual disease progression or regression, or that it may be based on the normal variation between repeat CT examinations. This is particularly important as small airways are the most important site for early obstructive disease in current and former heavy smokers, and measurements of small airways disease may prove important for the evaluation of therapy or in monitoring disease progression in the early stages of smoking-induced lung disease. Unfortunately, quantitative air trapping measures on low-dose CT of heavy smokers showed considerable variation on repeat CT examinations, regardless of lung volume correction or breath hold reproducibility.

Regarding the longitudinal application of CT air trapping assessment, our results lead to the following considerations. First, uncorrected EXP_−856_ shows large variability on repeat CT examinations, whereas the variability is considerably less for uncorrected E/I-ratio_MLD_. Second, EXP_−856_ is more sensitive to the expiratory effort, which is illustrated by the significant difference in the limits of agreement between the subgroups with superior and inferior breath hold reproducibility on repeat CT examinations. Third, application of lung volume correction does not significantly narrow the limits of agreement of the CT air trapping measures. An interesting finding is that application of lung volume correction can create negative values for EXP_−856_, while it is expressed in percentages (see Table 3). This may well be explained by the fact that the volume correction method used assumes a simple linear relation [17, 18], which seemingly does not apply for expiratory analyses. Application of the volume correction method in E/I-ratio_MLD_, which combines inspiratory and expiratory data, does not encounter these difficulties. Taken together, our results suggest that E/I-ratio_MLD_ is the preferred CT air trapping measure, because it shows the narrowest limits of agreement and is least dependent on variations in inspiratory and expiratory volume. Nevertheless, our main conclusion is that both CT air trapping measures may not be very suitable for longitudinal application, given the substantial variation between CT examinations regardless of good lung volume reproducibility or lung volume correction. Whether other lung volume correction methods or exact replication of lung volume by spirometric gating sufficiently improves variation between CT examinations requires further study.

The strength of our study is that it provides novel information on the variation in CT air trapping measures on repeat CT examinations. Further, our study population was imaged on the same CT system with the same protocol at both visits, and additional quality control using a phantom was performed to ensure constant CT numbers over time. This approach eliminates many confounding factors on the variation on repeat quantitative CT measures.

Our study also has limitations. Owing to the strict exclusion criteria our study population is fairly small. However, these strict criteria are needed to obtain valid results on variation in quantitative CT measures on repeat examinations. Another limitation is the follow-up period between the two CTs. On the basis of pathophysiological knowledge that air trapping in smokers is an expression of remodelling and obliteration of the terminal bronchioles [23], we assumed smoking-induced small airways disease in our population-based cohort with no or only mild COPD to be stable in our short follow-up period. Nevertheless, a repeat acquisition directly following the initial CT would be more ideal to eliminate possible differences over the period of 6 weeks. Further, it must be noted that this study used low-dose CT data, which might limit the generalizability to standard-dose acquisitions. Finally, we used a breath hold instructions instead of spirometric gating, which may improve agreement between repeat CT examinations. However, nearly all CTs are obtained without respiratory gating [4, 5, 24] and the present study therefore provides data that apply best to today’s common practice.

In conclusion, this study reports the variation in quantitative CT assessment of air trapping on repeated low-dose, non-gated CT examinations. Although the E/I-ratio_MLD_ seems preferable over EXP_−856_, we found considerable variation for both methods regardless of lung volume correction or proper lung volume reproducibility. Our findings imply that the evaluated quantitative CT air trapping measures may not be suitable for longitudinal application using current techniques.
